# Island biogeography, competition, and abiotic filtering together control species richness in habitat islands formed by nurse tree canopies in an arid environment

**DOI:** 10.1080/19420889.2022.2139471

**Published:** 2022-11-01

**Authors:** Ali A. Al-Namazi, Stephen P. Bonser

**Affiliations:** a Evolution and Ecology Research Centre, School of Biological, Earth and Environmental Sciences, UNSW Australia, Sydney, Australia; b King Abdulaziz City for Science and Technology (KACST), Riyadh, Saudi Arabia

**Keywords:** competition, facilitation, island of fertility, island biogeography, nurse plant, plant–plant interactions

## Abstract

The theory of island biogeography predicts that island size is a key predictor of community species richness. Islands can include any habitat surrounded environments that are inhospitable to the resident species. In arid environments, nurse trees act as islands in an environment uninhabitable to many plant species, and the size of the canopy controls the size of the understory plant community. We predicted that plant species richness will be affected by the area of the habitat and decrease with habitat isolation. We sampled the adult and seedling plant communities at canopy center, canopy edge, and outside canopy microhabitats. We found that species richness in both adult and seedling communities increases with increasing island area. However, richness in seedling communities was greater than in adult communities, and this effect was greatest at the canopy center microhabitat. Competition has been demonstrated to be more important in controlling species distributions near the canopy center, and stress is more important near the canopy edge. Thus, our results suggest that neutral forces, biotic interactions, and abiotic filtering act together to control species richness in these island communities.

## Introduction

Island biogeography theory predicts that species richness in a community on an island will be determined primarily by a combination of island size and isolation, [MacArthur and Wilson, [1]], [2]. Large islands are predicted to have more species than small islands since more species can support populations of viable size, and large populations are less likely to become extinct through random processes [3]. Islands that are close to a mainland or other islands are predicted to have greater species richness due to higher rates of colonization [4]. Island biogeography (and more broadly, neutral ecology) has been an influential ecological theory, potentially powerful in predicting patterns of community species richness [2]. Island biogeography assumes the demographic neutrality of species (i.e., species are essentially identical in their per capita probabilities of giving birth, dying, and migrating) to explain patterns of species richness, abundance, and distribution [2,5]. However, Island biogeography is also controversial as it neglects how differences between species contribute to community assembly [6].

Traditional ecological theory predicts that species responses to abiotic and biotic interactions are primarily important in controlling community assembly. Abiotic stresses can filter species out of a community if they are incapable of physiologically tolerating the environmental conditions within a community [7,8]. If an individual of a given species passes the environmental filters, intense competition [8] or interactions with enemies [e.g. predators or herbivores – 9, 10] can exclude species from a community.

Most studies on island biogeography tend to measure the richness as the adults of each species found already established on habitat islands and assess whether species richness across islands is consistent with biogeography predictions [e.g. 11–17, but see 18]. This is problematic since results that are consistent with island biogeography do not preclude the possibility that other processes control species richness in a community. Importantly, testing island biogeography predictions based on the richness of established individuals does not account for environmental filtering or biotic interactions acting on emerging juveniles that may remove species from a community prior to becoming established. This may be particularly important for plants, since plants disperse primarily as seeds and dispersal (migration) is central to island biogeography [19,20]. However, other ecological forces will determine the recruitment of seedlings into the community. For example, competition with species that already present on the island reduces seedling establishment and the colonization success of migrating species [18,21]. Thus, if small and/or isolated islands receive fewer migrant species and have lower seedling species richness than large and/or close islands, and competition removes a high and constant proportion of species across islands, then species richness on islands can be consistent with the predictions of island biogeography even though competitive exclusion is the dominant force structuring community assembly.

Island biogeography is not limited to terrestrial areas surrounded by water, the island could be any area that provides a benign environment for the survival and reproduction of species and is surrounded by unsuitable habitat [22]. In extremely stressful environments, islands under the canopies of nurse plants provide a suitable environment for growth and survival numerous of herbaceous species. Therefore, plant communities under nurse plants are functionally a series of islands where most species exist surrounded by a matrix of habitat beyond the physiological tolerance limits of these species [23–25].

In arid environments, the communities under nurse plants assemble in microhabitats depending on abiotic stresses and the facilitative impact of nurse trees in these microhabitats, and the productivity of microhabitat [26,27]. The impact of facilitation by nurse plants is high in the microhabitat at the center of the canopy, moderate in the microhabitat at the edge of the canopy, and low in the microhabitat outside the canopy. Therefore, on these islands, there is a mix of species that exist primarily in the least stressful environments (canopy center), those that exist in moderately stressful environments (canopy edge) and some species in high-stress habitats outside nurse plant canopies. In a previous study, we demonstrate that within-guild competition structures the herbaceous understory community in the least stressful environment (center of the canopy) while stress tolerance and within-guild facilitation between species under the canopy structures the herbaceous community in the more stressful environment at the edge of canopy [26].

In plant communities, the richness of seedlings emerging from the seed bank represents the offspring of species already present in the community plus those arriving from other communities. Differences in species richness of emerging seedlings and established (adult) plants can be used to test for a reduction in richness between seedling communities and adult communities due to environmental filtering or biotic interactions. We examined seedling and adult communities in canopy center (habitats where within-guild competition is important in structuring communities) and canopy edge (habitats where stress tolerance and abiotic filtering is important in structuring communities) microhabitats [26]. Thus, differences between canopy and edge habitats in seedling and adult species richness relationships can be important in assessing the importance of environmental filtering and biotic interactions in controlling community species richness. We examined these effects in an arid environment within a reserve in Saudi Arabia. We tested the following predictions: 1) Species richness will increase with the area of the habitat and decrease with habitat isolation (i.e. patterns of species richness will be consistent with the predictions of island biogeography). 2) Species richness in these island communities will be also controlled by abiotic filtering and competition. Thus, the interaction between neutral biogeography and ecological forces will control community species richness in these communities.

## Methods and Materials

### Island measurements

The genus *Acacia* is widely distributed in several ecosystems [28]. *Acacia gerrardii* (Benth.) is one of *Acacia* species that widely spread in the arid environments, and is a dominant tree in the many of the arid regions in Saudi Arabia. We selected ten trees representing a range of canopy areas for this study. The canopy diameter of these trees varied between 6 and 14 meters, and habitat areas under the canopies ranged between 48 and 146 m^2^. The coordinates of each *A. gerrardii* tree were determined by GPS (GARMIN GPSmap62). Then, the locations of 10 *A. gerrardii* trees were determined by Google Earth Application by using their coordinates. The isolation of the target *A. gerrardii* trees was measured as the distance between the island and its nearest neighbor of minimum canopy size 24 m^2^. Canopies less than 24 m^2^ found not to have a nurse plant effect and do not support understory communities. Therefore, the small trees with a canopy size of less than 24 m^2^ have been excluded. Distances between canopies were estimated using the ruler function on Google Earth.

### Vegetation sampling

We sampled the vegetation communities under nurse tree canopies near the end of the wet spring season in 2013 when the herbaceous community was at its maximum density. We sampled three microhabitats: at the center of the canopy of *A. gerrardii* (within 3 m of the tree stem), at the edge of the canopy (within ±50 cm of the canopy edge), and outside canopy (5–10 m away from the canopy). Two transects of three quadrats of 1 m^2^ were selected in each of three microhabitats (2 quadrats under, 2 at the margin and 2 outside the canopies of individual of *A. gerrardii*) in total of six at each tree, in two directions (North and South) to avoid the impact of shade extension in the morning and afternoon in the west and east directions, respectively.

The mean abundance of species (the number of individuals per species), Species density (the total number of species occurring per (1 m^2^) unit area), and plant cover (the percentage of occupied area by a plant species in (1 m^2^) unit area) were recorded in all quadrats in each microhabitat (under, at the margin and outside the canopy).

### Seedling sampling

Three samples of soil (30x30cm and depth 5 cm) were haphazardly collected from each of the three microhabitats under each of the *A. gerrardii* canopies. Samples were put in plastic bags and transferred to a lab. In the lab, samples were sieved to remove the stones and dried branches from the samples. Then they were placed evenly in germination trays (30 × 40 × 8 cm). In late May 2013, trays were placed in the glasshouse at approximately 25°C and watered with a fine spray from above (each tray was irrigated with 700 ml three times a week). Emerging seedlings were counted and discarded after identification. The seedlings which were difficult to be identified were counted, and some of them were transplanted and grown separately until the age where they can be identified. Seedling sampling continued until no further seedlings emerged from the sample. The seedling emergence observation took up to 10 weeks depending on the ability to define the species.

### Data analysis

Single factor analysis of variance was used to test for significant differences in seedling species richness between canopy center, canopy edge and open microhabitats. Tukey's HSD test was used to assess pairwise differences between means. We used simple linear regression to test for a significant relationship between species richness of established adult plants and island size and island isolation, separately. We then used a general linear model (GLM) to analyze the main effects of biogeography (the area of canopy), life stage (seedling versus adult), and microhabitat (canopy edge, canopy center, or outside the canopy) and the interactions between these effects on variation in species richness. Isolation was difficult to establish with certainty since there were small trees (trees less than 2 m canopy diameter) in the reserve that we did not include in the isolation analysis but can host small herbaceous communities. Further, unlike islands in the ocean, the non-island areas here could support some of the plant species, so islands were not completely isolated from other islands. Thus, we used the island (canopy) area as the primary neutral biogeography variable in the analysis. The effects of stage and microhabitat were fixed factors while the effect of island area was included as a random factor due to the random selection of trees [see 29]. The different effects of the general linear model can be used to assess the significance of the ecological factors that may control plant species richness. The area effect assesses the significance of biogeography on variation in species richness. The stage effect (seedling versus adult) assesses the significance of ecological effects (environmental filtering and competitive exclusion) on species richness. The microhabitat effect assesses the significance of different ecological processes (competition versus ecological filtering) on variation in species richness. All analyses were conducted using SPSS version 16.0 (SPSS Inc, Chicago, USA).

## Results

**Figure 1.**
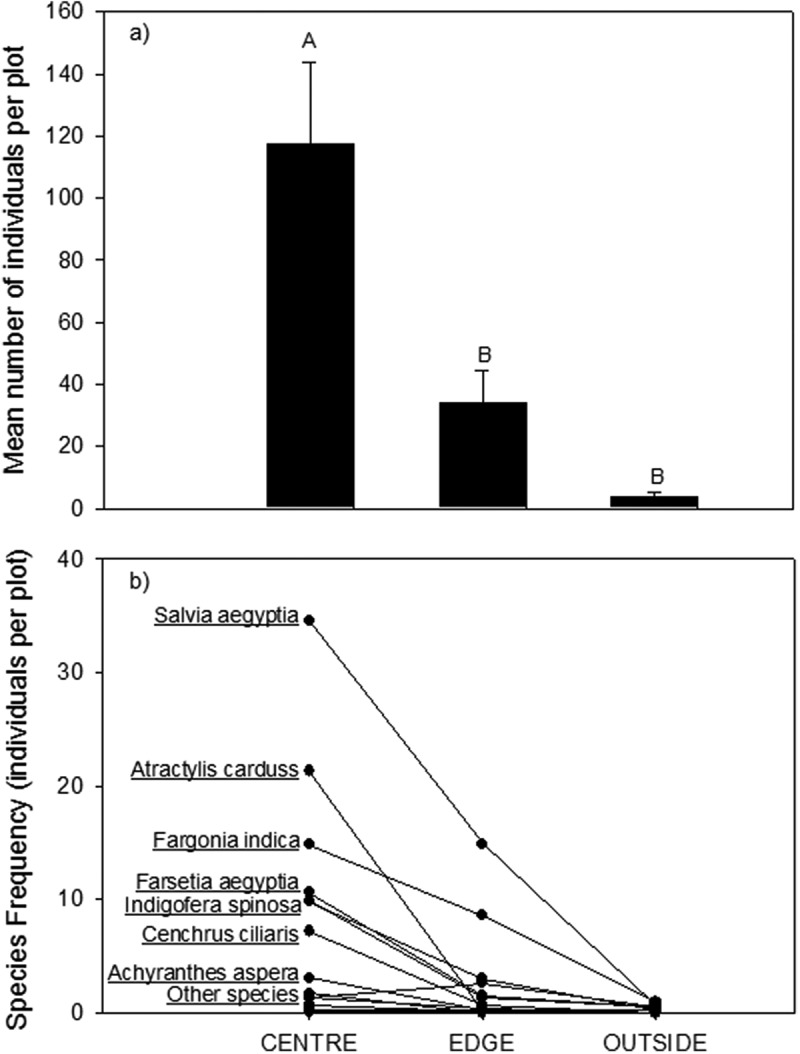
A) The mean number (±SE) of individual seedlings across all species emerging from soil seed bank in three different microhabitats (center of canopy, the edge of canopy and outside of canopy). b) The mean frequency of each species emerging from the seed bank in each of the microhabitats. Species with the highest frequencies are labeled on the figure. The species with low frequency (labeled as other species) were: *Senna italica* (the species with relatively higher frequency in the edge microhabitat), *Stipagrostis sp, Lycium shawii, Tribulus terrestris, commicarpus grandifloras, Dactyloctenium scindicum, Plantago sp, Launaea mucronata, and Aizon canariense.*

The mean of total number of individuals of all species emerging from soil seed bank and the frequency of these species are significantly higher in at the canopy center microhabitat than the abundance of species at the edge or outside the canopy (Figure 1). Species richness and the abundance of many species decrease with increasing distance of the microhabitat to the center of the canopy.

**Figure 2.**
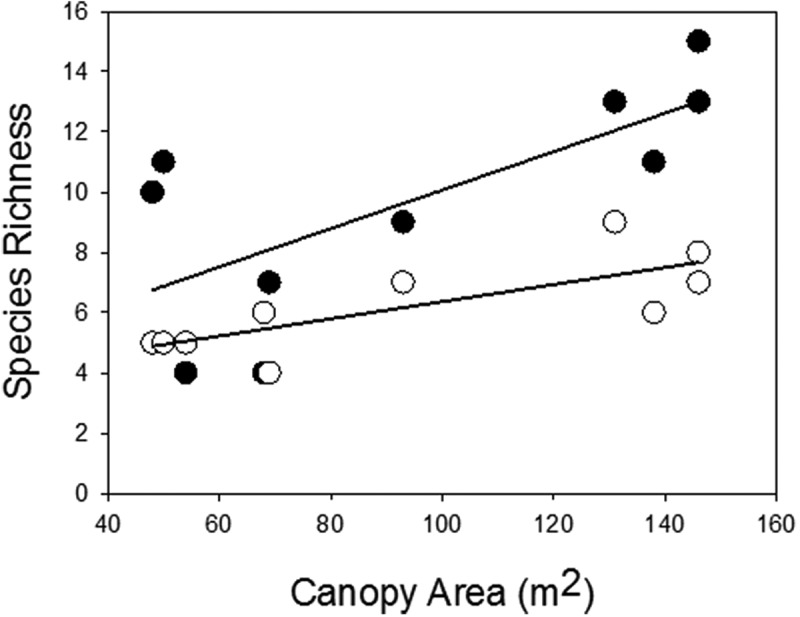
The relationships between island area and species richness in the soil seed bank (●) (regression: n = 10, r^2^ = 0.5, p = 0.021), and adult plants in the standing vegetation (○) (regression: n = 10, r^2^ = 0.59, p = 0.01).

We found a significant positive relationship between the number of herbaceous species emerging as seedlings from soil seed banks under the canopy of nurse plant and the number of species with established adult individuals, and the canopy island area (Figure 2). Moreover, there was a significant negative relationship between seed bank species richness and the isolation of the canopy (seedlings: slope = −0.09, n = 10, r^2^ = 0.65, p = 0.005; adults: slope = −0.03, n = 10, r^2^ = 0.4, p = 0.05).

**Figure 3.**
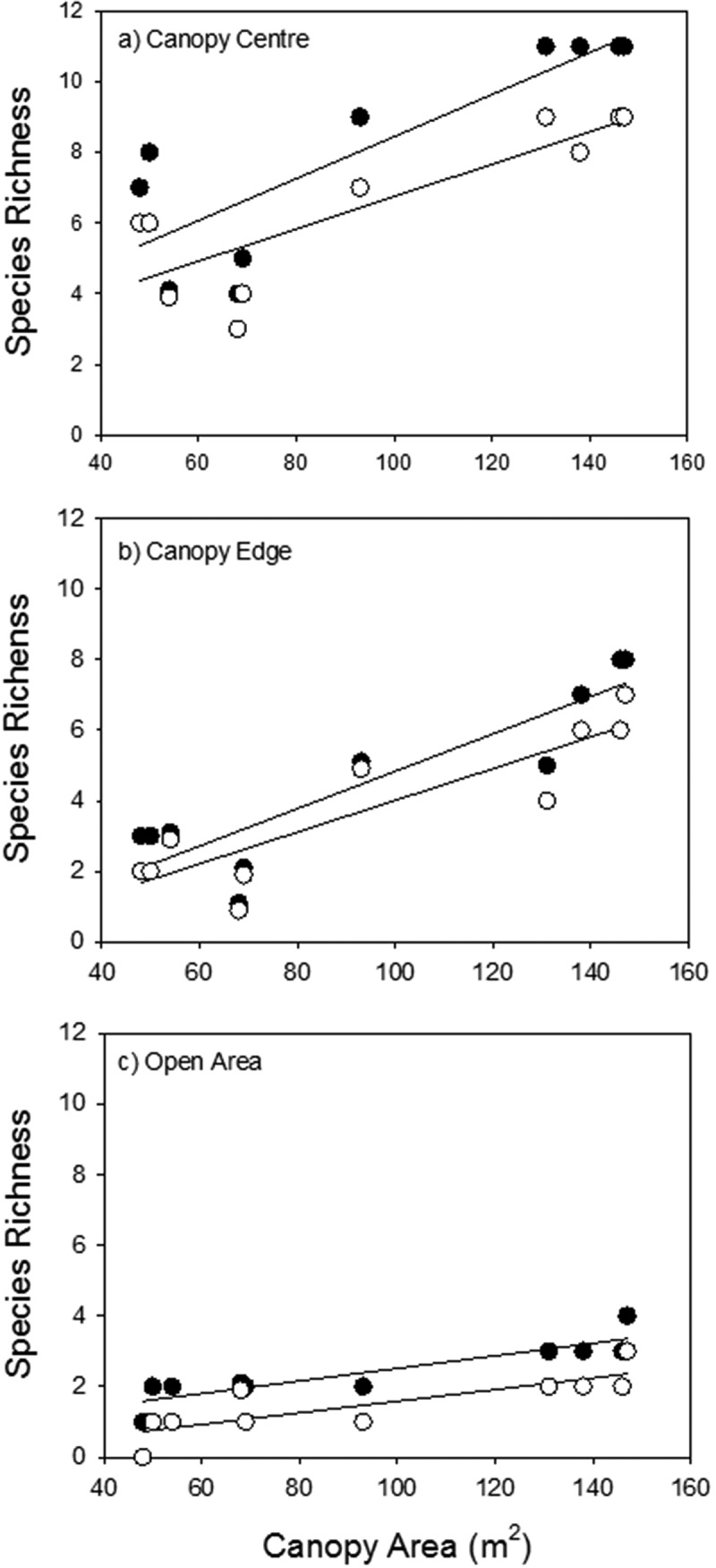
The relationships between island area and species richness in soil seed bank (●) and adult plants in the standing vegetation (○) in the three microhabitats along changes in the area of islands: a) at the canopy center (seedlings: n = 10, r^2^ = 0.71, p = 0.002; adults: n = 10, r^2^ = 0.70, p = 0.002); b) at the canopy edge (seedlings: n = 10, r^2^ = 0.80, p = 0.001; adults: n = 10, r^2^ = 0.78, p = 0.001); and c) outside the canopy (seedlings: n = 10, r^2^ = 0.79, p = 0.001; adults: n = 10, r^2^ = 0.60, p = 0.005).

Each of the main effects of island area, life stage and microhabitat had a significant effect in explaining variance in species richness. In addition, each of the two-way interactions, but not the three-way interaction, between the main effects was significant (Table 1). Larger islands had more species than smaller islands, and this effect was greater in seedlings than adult plants (Figure 2). The effect of island size on species richness was also greater at the canopy edge microhabitat, where the loss of species between the largest and smallest islands was about 70%. In contrast, the loss of species was about 50% at in the canopy center microhabitat (Figure 3). Species richness was lower in adult plants than for seedlings, and this loss in richness was greater at the canopy center than canopy edge microhabitats (Figure 3). Overall, the microhabitat was the main effect explained more variance than the other main effects, and the area x microhabitat was the interaction term explaining the most variance in species richness (Table 1).

**Table 1.** Mixed model analysis of variance results on the impact of stage (seedlings and adults), and microhabitat (center of canopy, edge of canopy, and open) as fixed factors, and area as random factor on plant richness.

Source of Variation	Sum of Squares	df	MS	P
Area (A)	162.2	8	15.4	0.001
Stage (S)	14.8	1	14.8	0.001
Microhabitat (M)	256.5	2	128.8	<0.0001
S x A	4.18	8	0.523	0.013
M x A	53.4	16	3.34	<0.0001
S x M	2.25	2	1.12	0.004
S x M x A	2.27	16	0.142	0.830
Error	1.5	6	0.250

Species richness outside the canopy in the open area was much lower than the canopy microhabitats, and species richness of the seed community was slightly higher than the established plant species richness. Outside the canopy, species richness tended to increase with increasing area of the adjacent canopy (Figure 3c)

## Discussion

Neutral theory (i.e. island biogeography) and ecological theories such as niche theory make contrasting predictions on the variation in community species richness, and the debate around these theories remains intense [5,30]. Many ecologists predict that plant community structure is driven fundamentally by the abiotic environment, interactions among species, and trade-offs in the performance of species across habitats [e.g. 31, 32], while others predict that plant community structure is determined mainly by the role of demographic stochasticity and neutral processes [e.g. 2, 33]. These arguments suggest that one of these two hypotheses is incorrect [e.g. 5]. Some recent studies suggest that niche and neutral process in species assemblage are integrated [34], and may involve an emergent neutrality mechanism [30]. In this study, we demonstrate that community species richness on islands is controlled by both neutral processes and fundamental ecological differences between species. However, these processes need not be integrated as they may act at different stages of plant life cycles.

Island biogeography plays a central role even with the very small islands and fragments (e.g. islands of fertility under the canopies of nurse plants). Although island edges are not perfectly defined, the vegetation pattern (both with emerging seedlings and adult plants) on these small islands adheres to the main principles of island biogeography theory (i.e., species–area and species–isolation relationships) [35, Losos and Ricklefs [36, 4, 37]. While our results for species richness of both seedling and adult communities remain consistent with island biogeography, the loss of species between seedling and adult stages suggests that communities assemble based on differences between species. Most studies (particularly on plants) examine richness long after ecological processes have a chance to act on community assembly (see introduction) – the resulting communities may assemble in a manner consistent with island biogeography, but there is no ability to assess the importance of other processes. Migration is a key factor in island biogeography [19,20]. Species richness of seeds in the soil represents the plant community after migration and prior to ecological sorting. The fact that we see higher seedling diversity in soil seedbank than adult or established plant diversity under the canopies suggests that some plant species are migrating from other communities. Further, the limitation of seed dispersal which is a key point for island biogeography theory [38,39], also plays a part in plant community structure in these islands as the seeds in soil seed bank decreases with increasing distance from the center of the island. However, results that are consistent with island biogeography do not exclude the possibility that ecological factors may impact species richness, or even be a dominant factor controlling community assembly.

Our results are consistent with the hypothesis that community species richness determined by environmental filtering and competition occurs within a biogeography framework. For example, we found that the species richness of seedlings emerging from soil seed bank was greater than the richness of standing vegetation species already established on these islands under the canopies of *A. gerrardii* trees. This means that the potential species representation in the seed banks (which is the sum of what was left by the resident species plus the migration from other islands) is higher than the number of species in the standing vegetation. The species in the seedling community assemble prior to any other ecological sorting or the effects of competitive exclusion among the coexisting herbaceous species and the sorting resulted from abiotic stress.

Plant community structure on these islands located under the canopies of *A. gerrardii* trees is driven by complex forces and structured by several ecological processes in addition to neutral island biogeography forces. For example, the persistence of plant communities is controlled by the facilitation by the nurse plant (*A. gerrardii*). These nurse plants, therefore, create islands of fertility under their canopies. The intensity of the facilitative impact of nurse plant varies among different microhabitats under its canopy [e.g. high facilitation at the center of the canopy and moderate facilitation at the edge of the canopy and low or no facilitation outside the canopy, 26]. The heterogeneity among these three different microhabitats is also consistent with island biogeography theory since no island is homogeneous. Habitats at or near the shore of an ocean island are generally quite different from habitats in the center of an island. The close microhabitat to shore (e.g. at the edge of canopy) is a different environment than away from the shore (e.g. at the center of the canopy). Furthermore, the plant community structure in this area (i.e., the island of fertility under the canopy) is driven by the environmental stress. For example, species richness is high in the less stressful microhabitat at the center of canopy compared with that in the more stressful microhabitats at the edge of canopy or that in the high stressful outside the canopy (e.g. Figure 3).

Finally and perhaps most importantly in these island communities, our results suggest that biotic interactions are fundamentally important in controlling community richness. The microhabitat effect explained more variation than area, and the stage x microhabitat interaction explained more variation in species richness than stage x area interaction (Table 1). Overall, these results suggest that the microhabitat effect may be more important than island biogeography effects in controlling species richness, and microhabitat broadly describes a shift from abiotic filtering and facilitation to competition from outside the canopy and the canopy edge to the canopy center microhabitats. Moreover, the stage effect (i.e. seedling versus adult species richness) was greatest at the canopy center, where competition is central in controlling populations. However, it is possible that the microhabitat effect explained more variation due to higher replication (each microhabitat was replicated 10 times, versus a total of 10 independent island habitats). The difference between the species richness of the standing vegetation and seedling emerging from the soil seed bank decreases with increasing the stresses across microhabitats (Figure 3). The large difference between the species richness of adults and seedlings in the center of canopy suggests that competitive exclusion is a key factor in controlling species richness in this microhabitat. Dominant species in the canopy center microhabitat tend to have higher competitive ability [26]. Stress also has a major impact in controlling species richness. In the edge microhabitat the abiotic stresses play a key role to structure the plant community, as stress sorting species, so only the plants that have the ability to tolerate stress can live, so become dominant in this microhabitat. However, the proportion of species lost at the canopy edge was higher than at canopy center. This could be explained by the fact that many species can survive competition but remain at very small sizes, while abiotic stress can kill quickly [40].

The tree island could be affected by several factors. The age of tree could affect the species richness, as the older trees which are also larger tend to accumulate more migrant seeds, and have more species [41,42]. Plant communities under older trees also have more time to accumulate species, thus older communities may be more species rich. However, the boundaries of island can be unclear, potentially making the size of island communities difficult to define. In our study, the nurse tree canopies define a clear boundary between low and high abiotic stress. Continuous and dense herbaceous vegetation can be found directly under the canopy, this vegetation becomes very sparse immediately outside the canopy. Thus, we believe the area of the canopy is a good measure of island area. Interestingly, the island area impacts species richness outside the canopies – species richness increases with canopy size in the outside canopy microhabitat (Figure 3c). This is likely due to greater propagule supply from communities under larger canopies. While some adult species persist in these open areas, most do not, and the areas outside the canopies are probably comprised mostly of unstable sink populations reliant on seeds from the nearby under-canopy herbaceous communities.

Our study contributes to resolving the debate between neutral and niche ecological theories [see 5], as we demonstrate that community assembly can be controlled by both processes. Community assembly is driven by the neutral ecology especially in the first stages (e.g. seedlings) and then community is controlled by the ecological sorting and competitive exclusion after the seedling stage. Further, the effects of competition and stress may be more important than neutral effects in controlling species richness in communities, at least in these relatively small island communities in a stressful arid habitat. Overall, the results of our study demonstrate that while community species richness can be consistent with the predictions of island biogeography, factors such as competitive exclusion and ecological sorting may be acting to structure these communities. Our research highlights the importance of examining the factors controlling species richness in communities after migration and during the recruitment of juveniles. Simultaneous effects of island biogeography and ecological factors may be common across communities.

These results advance an understanding of the complex processes that determine species richness in communities. Together neutral and ecological forces (competition, abiotic filtering) can control species richness. Numerous others in previous studies have advocated developing island biogeography to involve other ecological dynamics and concepts [43–46]. Indeed, aspects of habitat area and isolation are clearly important in contributing to species richness. However, these aspects of island biogeography are not necessarily neutral as they can influence the type and intensities of ecological interactions. Merging island biogeography and niche theory (and other ecological theories) offer an opportunity for a more complete understanding of species coexistence and the maintenance of diversity in ecological communities
